# Alternative Geometric Arrangements of the Nozzle Outlet Orifice for Liquid Micro-Jet Focusing in Gas Dynamic Virtual Nozzles

**DOI:** 10.3390/ma14061572

**Published:** 2021-03-23

**Authors:** Božidar Šarler, Rizwan Zahoor, Saša Bajt

**Affiliations:** 1 Laboratory for Fluid Dynamics and Thermodynamics, Faculty of Mechanical Engineering, University of Ljubljana, Aškerčeva 6, 1000 Ljubljana, Slovenia; rizwan.zahoor@fs.uni-lj.si; 2 Laboratory for Simulation of Materials and Processes, Institute of Metals and Technology, Lepi pot 11, 1000 Ljubljana, Slovenia; 3 Deutsches Elektronen-Synchrotron DESY, Notkestraβe 85, 22607 Hamburg, Germany; sasa.bajt@desy.de; 4 The Hamburg Centre for Ultrafast Imaging, Luruper Chaussee 149, 22761 Hamburg, Germany

**Keywords:** serial femtosecond crystallography, micro-jet, jetting, dripping, compressible two-phase flow, finite volume method, volume of fluid method, nozzle orifice design

## Abstract

Liquid micro-jets are crucial for sample delivery of protein crystals and other macromolecular samples in serial femtosecond crystallography. When combined with MHz repetition rate sources, such as the European X-ray free-electron laser (EuXFEL) facility, it is important that the diffraction patterns are collected before the samples are damaged. This requires extremely thin and very fast jets. In this paper we first explore numerically the influence of different nozzle orifice designs on jet parameters and finally compare our simulations with the experimental data obtained for one particular design. A gas dynamic virtual nozzle (GDVN) model, based on a mixture formulation of Newtonian, compressible, two-phase flow, is numerically solved with the finite volume method and volume of fluid approach to deal with the moving boundary between the gas and liquid phases. The goal is to maximize the jet velocity and its length while minimizing the jet thickness. The design studies incorporate differently shaped nozzle orifices, including an elongated orifice with a constant diameter and an orifice with a diverging angle. These are extensions of the nozzle geometry we investigated in our previous studies. Based on these simulations it is concluded that the extension of the constant diameter channel makes a negligible contribution to the jet’s length and its velocity. A change in the angle of the nozzle outlet orifice, however, has a significant effect on jet parameters. We find these kinds of simulation extremely useful for testing and optimizing novel nozzle designs.

## 1. Introduction

Serial femtosecond crystallography (SFX) [1] at X-ray free electron lasers (XFELs) is providing new capabilities for time-resolved macromolecular crystallography. In particular, it is revolutionizing the time-resolved and room-temperature structure determination of proteins. Although the samples get destroyed when interacting with high-intensity XFEL pulses, their diffraction patterns can be recorded before this happens. The XFEL pulses from the Linac Coherent Light Source (SLAC, Menlo Park, CA, USA) are delivered with a frequency of 120 Hz while the pulse frequency of the European XFEL (Schenefeld, Germany) can be as high as 4.5 MHz. When such X-ray laser pulses interact with the jet carrying the samples they produce shock waves, which can damage protein crystals and thus influence the data collection at MHz rates [2,3]. Hence, to avoid this the jets need to be very fast, with a velocity of 100 m/s or higher [4,5] to ensure exposing only fresh, undamaged samples. The natural environment of protein crystals is water or a buffer liquid. This is also why they are injected into the X-ray beam via a liquid jet. However, this liquid also produces unwanted background that often obstructs diffraction patterns from the sample. Therefore, the amount of liquid around the crystals has to be minimized, which means the jet diameter should be as thin as possible. Furthermore, the interaction between the jet and the intense XFEL beam should occur away from the nozzle exit. This is to protect the integrity of the 3D-printed plastic nozzles, which can be damaged by the intense X-rays. This favors longer jets. 

Recently, a comprehensive review compared different sample delivery arrangements for femtosecond crystallography [6]. The sample injection methods included liquid jets, mixing/double-flow jets, electrospinning, acoustic ejection, tape drive, support scanning, and sample delivery through gas-focused jets. The latter method is the most widely used. It is achieved with gas dynamic virtual nozzles (GDVNs) [7,8]. The application-dependent jet characteristics demand continuous investigation and development of new nozzle designs. The suitability of conventional and electrospray-assisted Rayleigh sources was investigated [9] but found to be limited. This resulted in rapid developments in GDVNs. Their manufacturing precision and complexity improved substantially once ceramic molding [10] and more recently 3D printing technology [11,12] was introduced.

**Figure 1:**
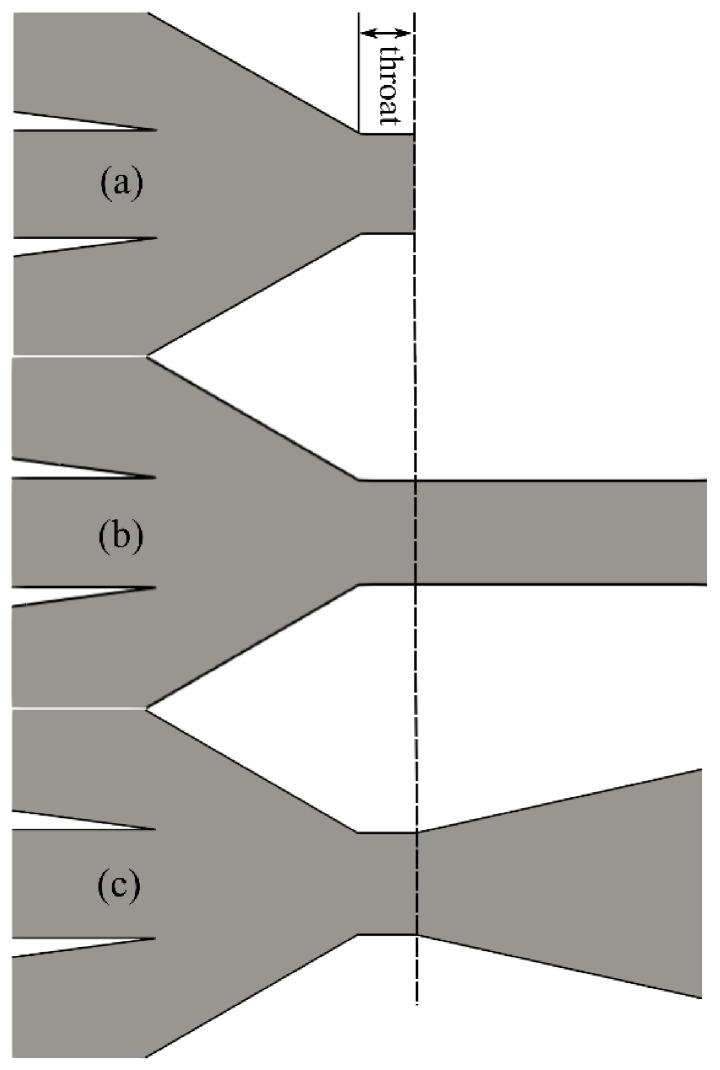
Scheme of different nozzle outlet orifices: (**a**) standard converging gas dynamic virtual nozzle (GDVN), (**b**) extended nozzle outlet orifice with straight channel, and (**c**) extended nozzle outlet orifice with diverging channel.

Experimentally validated numerical studies, including modelling of flow-focusing devices [13], jetting-dripping phenomena [14], primary breakup of jets with co-axial flowing air [15], and liquid sheets [16], for example, have established themselves as complementary to the experiments for studying micro-jet properties under different operating and design conditions. In the past, such modeling approaches [17] were limited to unrealistic incompressible gas-focusing behavior and jetting into the atmosphere. A few recent studies have, however, included compressible gas flow behavior in the nozzle simulations. They have coped with jetting into a low-pressure environment [18,19], the influence of GDVN geometry design [20], and the sensitivity of liquid [21] and gas [22] properties. The previously published GDVN designs used a standard converging gas capillary orifice as shown in Figure 1a.

In previous sensitivity studies with the standard [10] converging GDVN geometry, we tackled a wide spectrum of liquid and gas flow rates (Reynolds numbers from 50 to 70 and Weber numbers from 5 to 42, respectively), compared simulations with the experiments in terms of jet stability, diameter and length [19], changes of nozzle geometry [20], and use of different focusing gases [22], and predicted the influence of the micro-jet density, viscosity, and surface tension [21]. In this paper, we investigate alternative nozzle orifice designs and investigate their influence on the jet performance. It was previously observed [10] that, for SFX experiments, when delivering samples in a vacuum environment the conventional GDVNs operate with the choked gas flow. It is expected that if nozzle outlet geometry is changed, e.g., by adding channels of varying lengths (Figure 1b) or diverging angles (Figure 1c), then the liquid jet can be further accelerated. This motivated us to study how these modifications can influence the jet stability, its diameter, its length, and its velocity.

The modes of the liquid flowing from the GDVNs can be divided into the following stability conditions [23,24]: (a) an unstable meniscus and periodic ejection of drops (dripping), (b) continuous stable liquid thread, which finally breaks into the stream of droplets (jetting), or (c) spatially unstable jet, which whips laterally with some amplitude (whipping). For this study, stable jetting conditions are of particular interest.

In the following, we first describe the physical model and the solution procedure. Next, a study of 12 different nozzle outlet orifice designs is presented and discussed. Finally, the numerical model is verified with experimental data obtained from a jet produced by a 3D-printed nozzle with a converging–diverging nozzle orifice design. 

## 2. Materials and Methods

### 2.1. Problem Formulation

The cylindrical symmetry of the nozzles, gas flow, and liquid jet is considered throughout the present study. This assumption, together with the experimental evidence [19,25] that the micro-jets emerging from such nozzles mainly keep the axisymmetry before the breakup, allows building of an axisymmetric numerical model (*r-z*) instead of the full 3D model. 

This approach has previously been successfully assumed in similar numerical studies [14,20,25,26,27], with a proper account for the jet length, thickness, and velocity. The axisymmetric numerical model is unable to cope with the whipping instabilities [28] and the secondary breakup processes. Whipping instability is the lateral movement of the liquid jet away from its axis. Such instability occurs at high co-flowing gas flow rates and consequently influences the reliable interaction between the jet and the X-ray beam in SFX experiments. Both of these processes are outside the scope of the present study. However, the justification to neglect the whipping instability range is that it appears outside the range of liquid and gas process parameters that are used to achieve a stable jet.

A numerical model previously experimentally verified in terms of jet shape [19] and velocity [25] is also used in the present study. The model is based on a mixture formulation of Navier–Stokes equations for an immiscible gas–liquid two-phase Newtonian, compressible fluid. The finite volume method (FVM) discretization is considered [29,30], with the gas–liquid interface treatment using the algebraic volume of fluid (VOF) model [31]. The two-phase system is defined by a phase function $\alpha \left(x,t\right)\;$ at position x and time *t*, bounded between 0 and 1, that denotes the existence of one of the phases at the point $P\left(x,t\right)$:

(1) $$\alpha \left(x,t\right)=\left\{\begin{array}{cc} 1 & P\left(x,t\right)\in \;\mathrm{liquid}\\ 0 & P\left(x,t\right)\in \;\mathrm{gas} \end{array}\right.$$

The gas–liquid interface is considered in the points $P\left(x,t\right)$ with a discontinuous phase function. The phase function interface advection equation (without consideration of the phase-change) is given as:

(2) $$\partial \left(\rho \alpha \right)/\partial t+\nabla \cdot \left(\rho v\alpha \right)=0$$

where $v\left(x,t\right)$ and $\rho \left(x,t\right)$ represent velocity and density as a function of position x and time t.

The mass, momentum, and energy conservation equations in the mixture formulation are given as:

(3) $$\partial \rho /\partial t+\nabla \cdot \left(\rho v\right)=0$$

(4) $$\partial \left(\rho v\right)/\partial t+\nabla \cdot \left(\rho vv\right)=-\nabla p+\nabla \cdot \bar{\tau }+f_{\sigma }$$

(5) $$\partial \left(\rho e\right)/\partial t+\nabla \cdot \left(\rho ve\right)=-\nabla \cdot \left(p,v\right)+\nabla \cdot \left(\bar{\tau },v\right)-\nabla \cdot q$$

where $p\left(x,t\right)$ and $f_{\sigma }\left(x,t\right)$ represent pressure and surface tension forces, $\bar{\tau }$ is the viscous stress tensor, defined as $\bar{\tau }=\mu \left[\left(\nabla v\right)+{\left(\nabla v\right)}^{T}\right]-\frac{2}{3}\mu \left(\nabla \cdot v\right)I$, with μ and I standing for kinematic viscosity and identity tensor, respectively. Finally, e is a sum of the mass-specific internal and kinetic energies $e=c_{v}T+0.5{\left|v\right|}^{2}$, with $c_{v}$ and T standing for the specific heat capacity at constant volume and temperature, respectively. q=−k∇T represents the Fourier heat flux with the mixture thermal conductivity *k*.

Effective material properties of the mixture model are considered in Equations (6)–(10).

(6) $$\rho =\rho_{l}\alpha +\rho_{g}\left(1-\alpha \right)$$

(7) $$\mu =\mu_{l}\alpha +\mu_{g}\left(1-\alpha \right)$$

(8) $$k=k_{l}\alpha +k_{g}\left(1-\alpha \right)$$

(9) $$v=\left[v_{l}\rho_{l}\alpha +v_{g}\rho_{g}\left(1-\alpha \right)\right]/\rho$$

(10) $$c_{v}=\left[c_{v_{l}}\rho_{l}\alpha +c_{vg}\rho_{g}\left(1-\alpha \right)\right]/\rho$$

An ideal gas constitutive relation for calculation of the gas density is considered:

(11) $$\rho_{g}=p/\left(R_{g}T\right)$$

with $R_{g}$ denoting the specific gas constant.

The micro-dimensionality of the considered system and the presumed high speed allows the gravitational effects to be neglected. The surface tension force is determined by:

(12) $$f_{\sigma }=\sigma \kappa \nabla \alpha$$

with σ being the surface tension, and κ the curvature of the interface. The continuum surface model (CSF) [32] is used for the curvature calculation as $\kappa \left(\alpha \right)=-\nabla \cdot \hat{n}$, with $\hat{n}=\nabla \alpha /\left|\nabla \alpha \right|$ being the unit interface normal pointing from the gas to the liquid.

### 2.2. Computational Domain and Solution Setup

**Figure 2:**
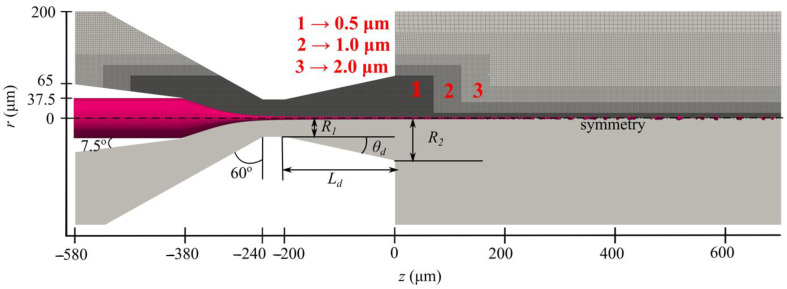
A part of the computational domain of a typical converging–diverging GDVN (below) and its discretization in the vicinity of the nozzle (above). *R_1_* = 35 µm is the fixed throat radius, *R_2_* and *L_d_* are the outlet radius and the length of the diverging channel, respectively. They are both varied in the present study. The total discretization domain has a radius of 1000 µm and a length of 3200 µm. The numbers 1, 2, and 3 mark the cell size and the areas of the mesh refinement.

The 3D-printed GDVN geometry shown in Figure 12 for producing liquid micro-jets possesses axial symmetry in the tip. As the numerical simulations were performed considering the only front tip part, an axisymmetric numerical model could be utilized. An axisymmetric solution setup with OpenFOAM (version 7, OpenFOAM Foundation Ltd, London, UK) was carried out, requiring a wedge-shaped 3D geometry. The wedge angle was selected to be equal to 4°, following the requirement of the optimum axisymmetric simulation setup [33]. The bottom plane of the wedge, in the present case, is the symmetry line. The size of the computational domain of the outlet chamber was the same as in our previous study [19] with a radius of 1000 µm and an axial length of 3200 µm. The domain was discretized with non-overlapping hexa-dominant cells, such that maximum refinement was provided in the region of expected large gradients of flow fields and phase function. The geometry of a representative nozzle and its discretization are shown in Figure 2, where the mesh refinement levels are represented by 1, 2, and 3, with cell sizes of 0.5, 1.0, and 2.0 µm, respectively.

Five patches (inlet liquid, inlet gas, walls, outlet, front, and back) were selected from the discretized domain on which the boundary conditions were applied. The mass flow rates of the gas and the liquid were used as inlet boundary conditions with the total pressure boundary conditions of 150 Pa applied at the outlet. A no-slip boundary condition was used at the fixed walls. The Dirichlet room temperature condition was used at the inlet, whereas at the walls and the outlet, the Neumann zero heat-flux boundary conditions were used. A special wedge-type boundary condition was required at the lateral sides (front and back) of the geometric patches. This boundary condition enforces a cyclic condition between the two patches. An overview of the applied boundary conditions is given in Table 1, with their detailed description and the numerical implementation found in [30].

The numerical model described in Section 2.1 was solved by a standard OpenFOAM solver (compressibleInterFoam ver.7). Detailed numerical implementation and user instructions are given in [30,33]. The compressible two-phase solver applied here uses the algebraic VOF method. Algebraic VOF causes the liquid–gas interface to diffuse over a few cells, which is countered by an artificial interface compression counter-gradient approach [34]. Additionally, the boundedness of α was ensured by using a multidimensional universal limiter for an explicit solution (MULES). Standard Gaussian finite volume integration [35] was used to obtain a higher integration accuracy of the derivative terms. A second-order *vanLeer* total variation diminishing (TVD) scheme [36] was used for the discretization of the convective terms. The cell-face interpolation of the velocity field was carried out by using a second-order TVD scheme *limitedLinearV* [30]. The time integration was performed by the backward differencing method [37]. The pressure velocity coupling was obtained by using the PIMPLE algorithm, where an adaptive time-stepping approach is used, ensuring the Courant number [38] $\mathrm{Co}=\;\left(\left|v\right|\Delta t/\Delta x\right)\leq 0.25$.

To ensure mesh-independent results, a related comprehensive study has previously been detailed for a similar injection molded GDVN [19]. It was carried out with the minimum cell sizes of 1.0, 0.50, and 0.25 µm with 90,000, 150,000, and 300,000 cells, respectively. A well-known Richardson extrapolation approach [39] was also utilized for a convergence check. The study was based on the consideration of the shape of the jet, which includes its diameter and length along with the flow fields at various cross-sections in the nozzles, as convergence criteria. It was observed that a reasonably converged solution can be obtained with 0.5 µm cells. The same spatial discretization density is used in the present paper.

Deionized water and helium gas were considered as focused and focusing mediums, respectively. Representative material properties of both mediums at standard temperature and pressure are given in Table 2. The numerical simulations start at *t* = 0 s with the liquid capillary initially filled with water.

The numerical simulation with the aforementioned computational model requires ~250 hours of CPU time to calculate 0.9 ms of real-time on 64 Intel (R) Xeon (R) processors. The numerical results were recorded at an interval of 1µs and consequently analyzed with ParaView [40] and Matplotlib [41].

## 3. Results

The considered GDVN micro-nozzles operate in choked-flow mode [10]. The expansion of the gas in the outlet chamber causes an accelerated gas flow at the nozzle outlet [19]. This type of liquid jet focusing, with free expansion, has previously been successfully utilized to focus high-speed jets [18]. The liquid jet is accelerated and focused mainly through a shear contact with the gas. A higher gas velocity due to expansion applies a higher shear force. However, the expansion of the gas in the vacuum environment rarifies it, consequently reducing the resulting shear force at the same time. We considered the modifications of the nozzle orifice geometry by 12 geometrical variations, listed in Table 3. They comprise (i) constant diameter channels of varying lengths and (ii) diverging channels of varying diverging angles. The constant diameter prolonged nozzle orifices were chosen to enforce an extended exposure of the liquid jet to the focusing gas. The diverging orifice of the nozzle was chosen since the choked gas flow in the nozzle opens the possibility of additional acceleration of the gas due to the converging–diverging supersonic acceleration of the choked flow [42].

The listed geometrical variants of the orifice in Table 3 could enhance the momentum harvesting from the co-flowing gas to the liquid as well as counter the instability waves on the surface of the jet while accelerating it.

**Figure 3:**
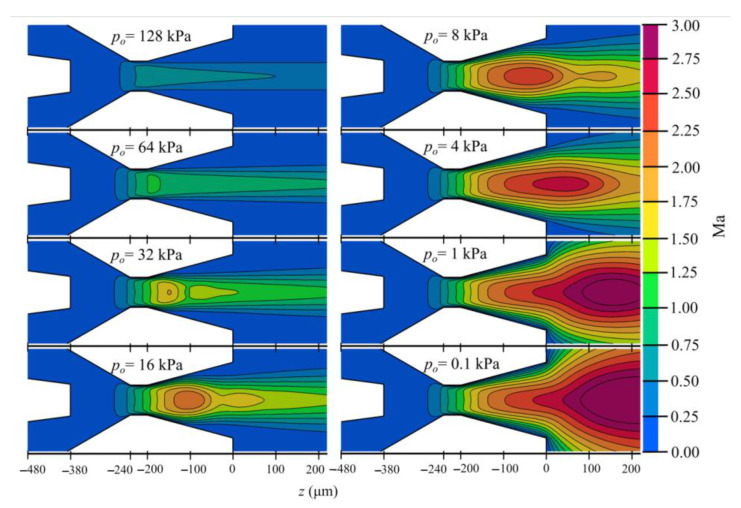
Mach number contours of the gas flow inside a nozzle with *L_d_* = 200 µm and *θ_d_* = 15^o^, for different outlet chamber pressures.

**Figure 4:**
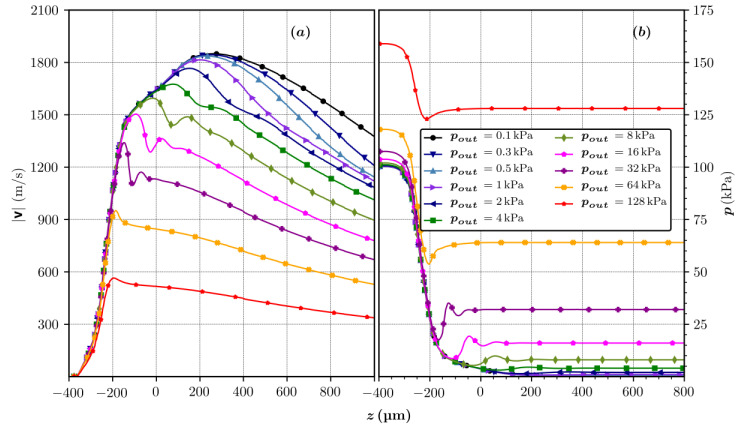
(**a**) Velocity and (**b**) pressure profiles along the symmetry axis of the nozzle with *L_d_* = 200 µm and *θ_d_* = 15°, as a function of the outlet chamber pressures.

With the addition of diverging outlet geometries, a possibility of shock appearance exists inside the nozzle, influencing the stability of the jet. This might be a major downside for such an approach. To analyze the described situation, a converging–diverging nozzle (Geometric variant 9 in Table 3) was simulated with an inlet gas mass flow rate of 22 mg/min and different outlet chamber pressures, as shown in Figure 3 and Figure 4. The Mach number maps in Figure 3 were calculated with $\left|v\right|/v_{s}$, where $v_{s}$ is the speed of sound in helium gas, with helium gas constant R=2077 J/kgK and specific heat ratio γ=1.66.

It was observed that at the highest outlet pressure of 128 kPa, the nozzle operation is subsonic. With a decrease of outlet pressure from 128 kPa to 64 kPa, the Mach number in the throat region reaches 1 and the nozzle gas flow becomes choked. A further decrease in the outlet pressure causes the shock wave to appear right at the start of the diverging section. Its position moves forward towards the end of the nozzle with the pressure decrease. At *p_o_* = 0.5 kPa, the nozzle design condition is reached, which is a shockless smooth expansion. The discussed operational conditions and the position of the shock waves are illustrated with pressure and velocity profiles along the symmetry of the nozzle in Figure 4. With the intended outlet vacuum pressure lower than 0.15 kPa, it becomes obvious that the jet focusing will not be disturbed by any interference from the shock waves.

**Figure 5:**
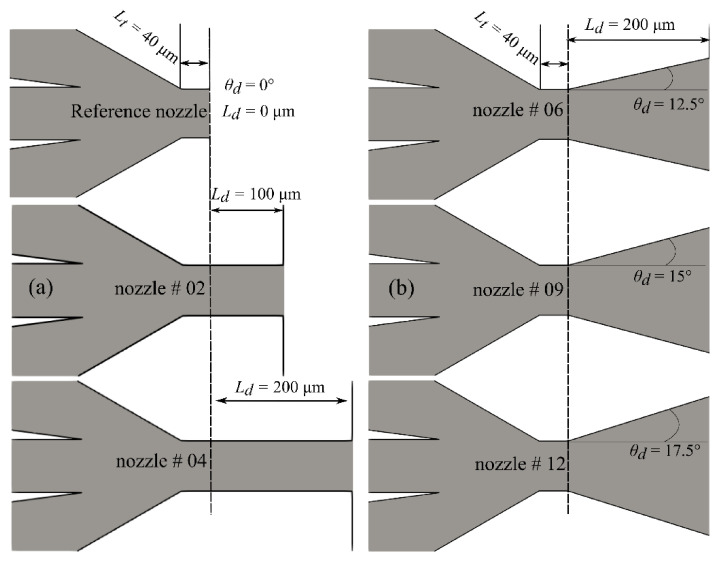
Scheme of the nozzles for 6 selected outlet orifice designs from Table 3: (**a**) zero diverging angle with different extending lengths, (**b**) constant diverging length of 200 µm with varying diverging angles.

The length of the outlet throat was varied from the nominal of 40 µm [19] to a maximum of 250 µm while keeping the angle at 0^o^, to investigate its influence on jet formation, its stability, and the acceleration (Figure 5a). In Figure 5b the length of the diverging part was kept constant at 200 µm and the diverging angle was increased from 12.5° to 17.5°. A range of geometric schemes is consistent with the data from Table 3. 

A fixed gas mass flow rate of 22 mg/min and a liquid flow rate of 50 µL/min were used in the simulation of all nozzle orifice variants. The related jet Reynolds numbers Rej=ρlQl/πRjμl ranged from 280 to 420 and Weber numbers $\left({\mathrm{We}}_{j}\;=\;\rho_{l}Q_{l}^{2}/\pi^{2}R_{j}^{3}\sigma ;\;\;R_{j}\;=\;D_{j}/2\right)$ from 270 to 360. The selected gas flow rate assured the choked flow operation at 150 Pa outlet pressure.

In the following, our study was focused on the changes in the nozzle orifice geometry while keeping the described operating conditions and the rest of the nozzle geometry fixed.

It was previously demonstrated by [27], through scaling analysis for a plate-orifice nozzle, that there exists an optimum geometric arrangement Hopt/D=0.6Dl/D14 ensuring a stable jet at a minimum liquid flow rate estimated for a given liquid capillary diameter *D_l_* and a nozzle outlet diameter *D* as Qmin/D=2.5Dl/D13μl/ρl. This approach to nozzle optimization was later successfully confirmed for ceramic injection molded GDVNs [20]. On either side of the optimum value of capillary-to-orifice distance (*H_opt_*), the minimum liquid flow rate *Q_min_* required to ensure the jet stability increases. The jet stability increases at the cost of liquid flow rate [10] for *H > H_opt_*, while for *H < H_opt_* flow-focusing shifts towards flow-blurring [43]. It has been common practice [10] for SFX experiments to design nozzles with larger *H* distances, to gain extra jet stability, while ensuring larger jet lengths. The nozzles considered in the present investigation have an *H* distance of more than twice the value of *H_opt_* predicted by [27]. They require a higher liquid flow rate than the minimum required to gain a stable jet. Additionally, the higher value of the liquid flow rate than the minimum required (as per scaling laws) provides thicker and longer jets, making it easier for visualization and investigation of the influence of the orifice variations. A nozzle design described in [19] was considered here as a base design.

The stability of the liquid meniscus acts as a precursor to the overall stability of the liquid jet. The stability of the meniscus is influenced by the instability waves traveling upstream due to the drop pinch-off at the tip of the jet and the strength of the energy sink due to the recirculation at the meniscus region.

**Figure 6:**
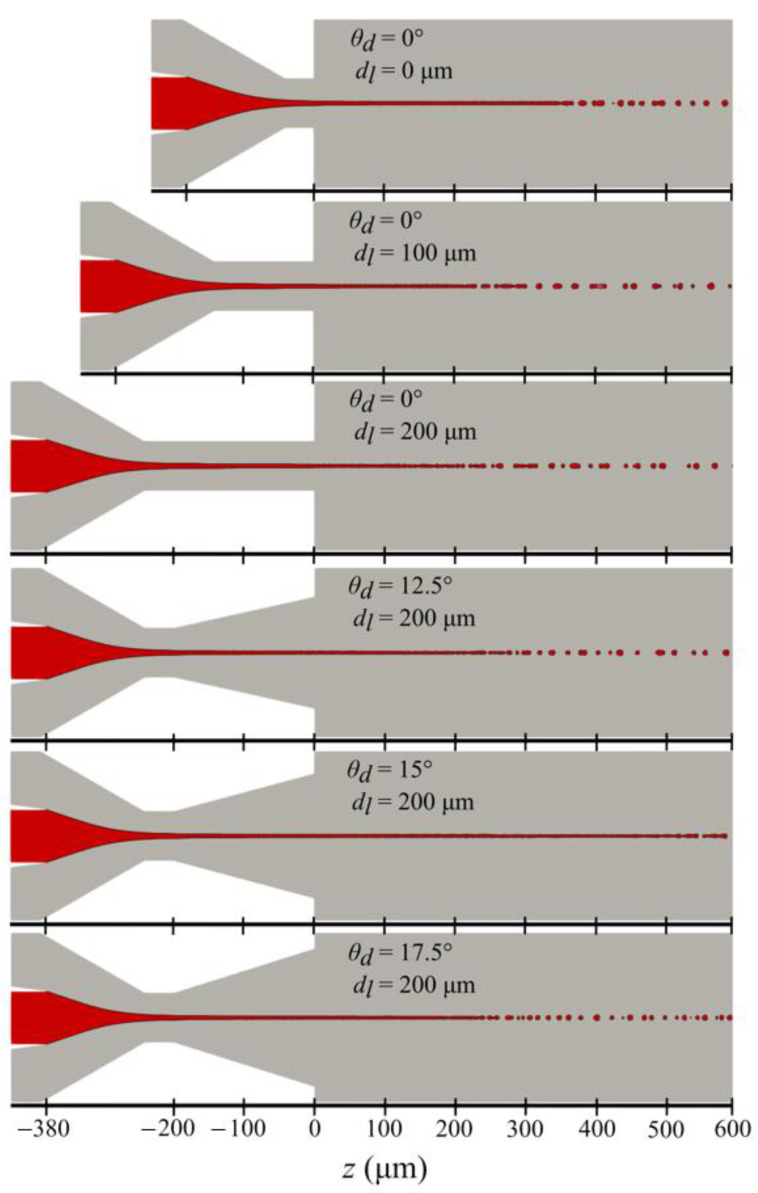
A plot of the jet shape at 0.8 ms for selected 6 nozzle outlet orifice variants from Table 3.

**Figure 7:**
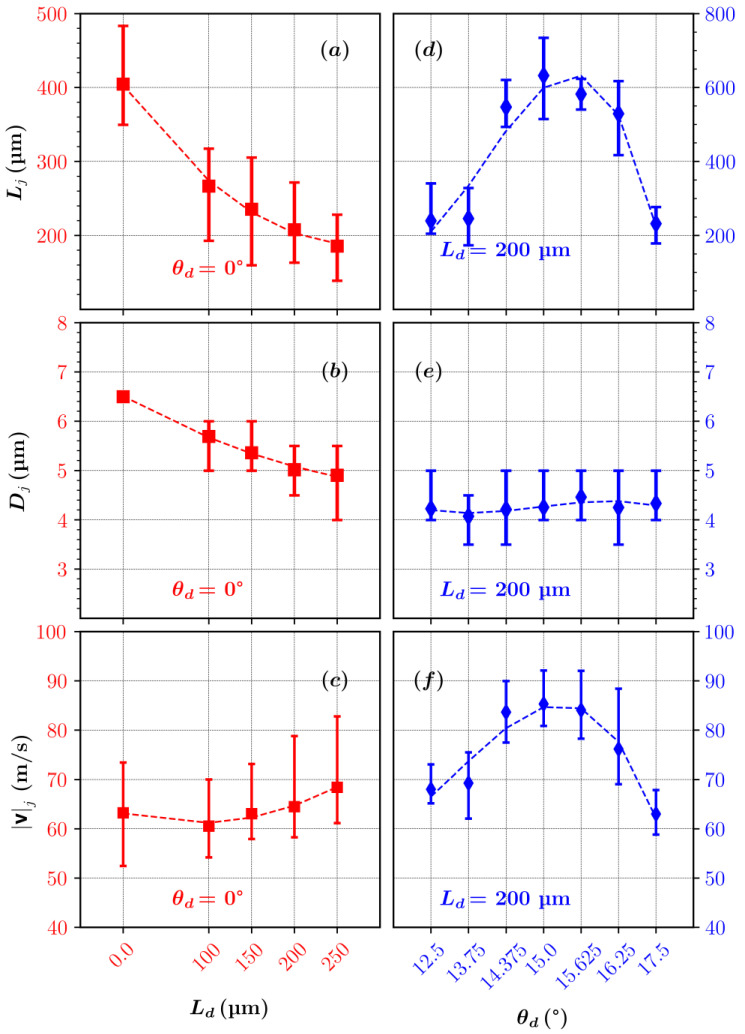
(**a**) Jet length, (**b**) diameter, and (**c**) velocity for 0° diverging angle and different nozzle outlet channel lengths, and (**d**) jet length, (**e**) diameter, and (**f**) velocity for different diverging angles and 200 µm of nozzle outlet channel length. The represented averages are calculated over 0.1 ms of simulation time and the error bars represent the minimum and maximum values occurring over averaging interval.

The set of nozzle geometric parameters within the ranges presented in Table 3 were analyzed in terms of the length, diameter, and velocity of the liquid jet. The jet length was measured from the nozzle outlet to the position of the first drop detachment, the diameter at the nozzle outlet, and the velocity at the tip of the jet. The influence of the nozzle orifice variation on these parameters is shown in Figure 6 and Figure 7.

The addition of the straight channels of increasing lengths (from 0 µm to 250 µm) at the nozzle outlet provides shorter (~400 µm to ~200 µm), thinner (~6.5 µm to ~5 µm), and faster (~63 m/s to ~68 m/s) jets.

It is indicated that the elongated orifice increases the flow resistance through it [44], causing the flow to lose strength in longer channels. It has previously been documented [45] that the viscous shear of the co-flowing gaseous phase determines the flow morphology of the focused medium. The same operating flow rates and the resulting velocities thus imply a smaller shear force towards the exit of the elongated orifice channel. A jet issuing from a prolonged channel does not have an increased shear damping in the outlet chamber required to counter the instability waves. This causes the jet length to be comparatively shorter than the basic nozzle design. This is also due to the fact that the jet becomes more prone to instabilities due to a larger distance from the meniscus.

Since the pressure drop increases with a larger channel length, a comparatively stronger sudden expansion at the outlet of the nozzle helps the jet to be slightly more compressed and accelerated.

In Figure 6 and Figure 7, an interesting trend in jet length, diameter, and velocity is observed with diverging channels of varying angles between 12.5° to 17.5°. When the constant diameter channels are replaced with the 200-µm-long channel of increasing diverging angles (from 12.5° to 17.5°), the resulting jets increase in length from ~200 µm with 12.5° to 650 µm with 15°. However, with a further increase in diverging angle to 17.5° the jet length decreases again to ~250 µm. A similar trend is observed with the jet velocity. The jet velocity first increases from ~65 m/s at 12.5° to the highest value of 85 m/s with a 15° diverging angle and then decreases to ~63 m/s with a 17.5° diverging angle.

The diverging angle of 15° provides the longest and the fastest jet. This finding is also consistent with the optimization of the converging–diverging nozzles in terms of the diverging angles on much large scales [46]. Ande and Yerraboina [46] also found out that a diverging angle of 15° tends to provide the strongest flow streams in the axial directions.

**Figure 8:**
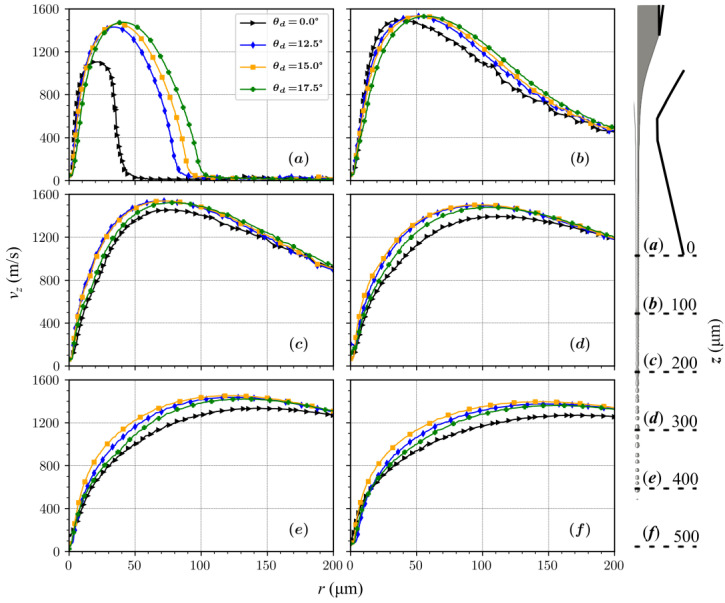
Axial component of velocity profile as a function of the radius at 6 axial positions for *L_d_* = 200 µm and diverging angles of 0°, 12.5°, 15°, and 17.5°.

**Figure 9:**
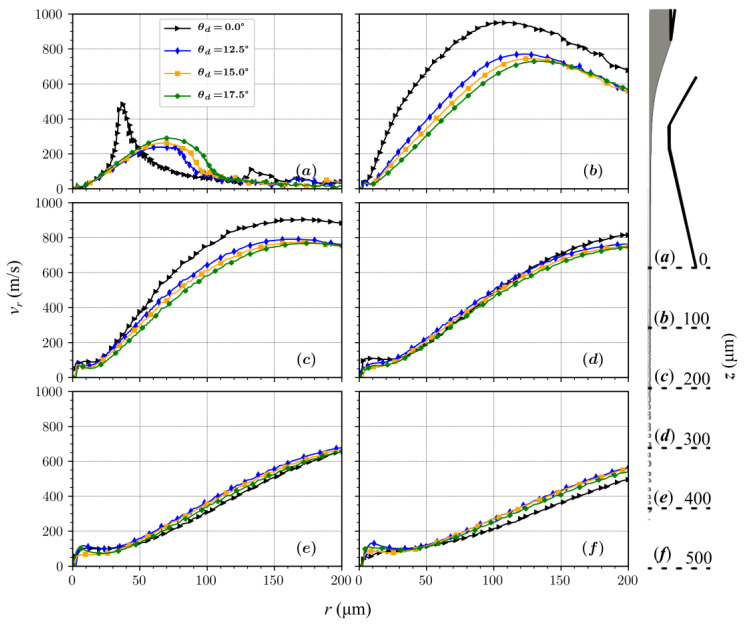
Radial component of velocity profile as a function of the radius at 6 axial positions for *L_d_* = 200 µm and diverging angles of 0°, 12.5°, 15°, and 17.5°.

Interestingly, the jet diameter at the end of the diverging part (*L_d_* = 200 µm) shows no significant change. This is attributed to the minimal changes in the flow fields at the nozzle outlet in the vicinity of the jet for all the diverging angles (Figure 8 and Figure 9). The difference in the velocity field for different diverging angles only becomes significant downstream from the nozzle outlet.

The radial component of the velocity at the nozzle exit remains almost unaffected when increasing the diverging angle from 12.5° to 17.5°. This causes the jet diameters, calculated at the outlet, to be similar for all diverging orifice geometries (Figure 7). When velocity streams expand in the outlet chamber, the axial component of velocity for a 15° diverging angle increases with the axial distance from the outlet, while for 12.5° and 17.5^o^ it decreases. The intensity of the gas expansion in the axial direction directly influences the amount of shear generated at the surface of the jet, consequently affecting the shapes and the velocities of the liquid micro-jets.

When compared with the converging–diverging nozzles in Figure 8 and Figure 9, the nozzles with constant outlet diameter channels do not sustain expansion strengths for larger axial distances. Such sustained velocity fields over larger distances are necessary to stabilize and accelerate jets. A stronger expansion in the radial direction appears at the nozzle outlet, which dissipates quickly at a distance of ~500 µm. An optimal flow for stabilizing the jet over a larger length needs to be stronger in the axial direction. When looking at different diverging angles in Figure 8, the optimal required flow happens with the nozzle having a 15° diverging angle. 

**Figure 10:**
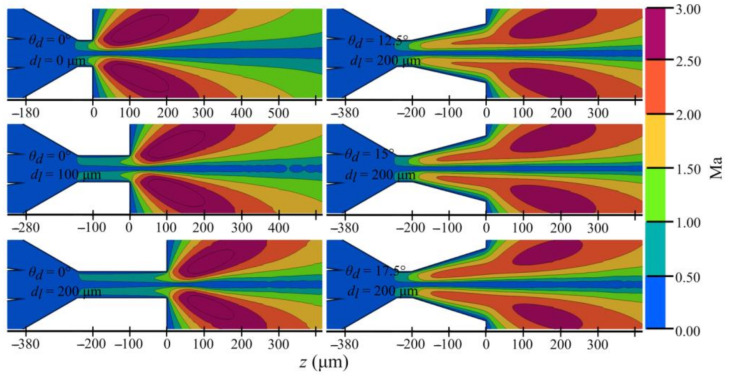
Mach number maps for selected 6 nozzle outlet orifices from Table 3.

Maps of Mach number in Figure 10 show how expansion profiles form in various nozzle geometries. It is seen that the maximum expansion strength for constant-diameter outlet channels is right at the exit of the nozzle. However, in the case of diverging nozzles, the expansion strength region extends over a larger axial distance.

**Figure 11:**
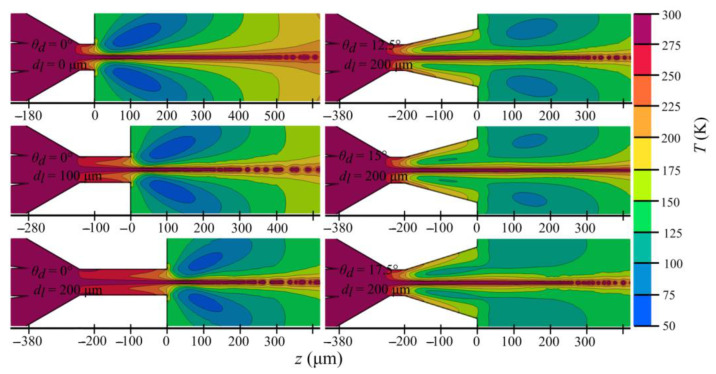
Temperature maps for selected 6 nozzle outlet orifices from Table 3.

Although strong expansion is beneficial for stabilizing and accelerating the jets, it can also result in drastic cooling of the gas (by ~200 K) in the outlet chamber (see Figure 11). However, even though the outer co-flowing gas experiences considerable cooling, the temperature distribution profiles in the vicinity of the jet show no significant change of the temperature inside the liquid jet. This is consistent with previous experimental [10] and numerical results [18,19]. This phenomenon can be explained by the presence of an envelope of a low-density gas, acting as an insulator of the liquid rather than as a coolant. Furthermore, due to high velocities, the contact time between the gas and liquid is too short to establish significant heat transfer.

### Comparison of Numerical Model with the Experiment

**Figure 12:**
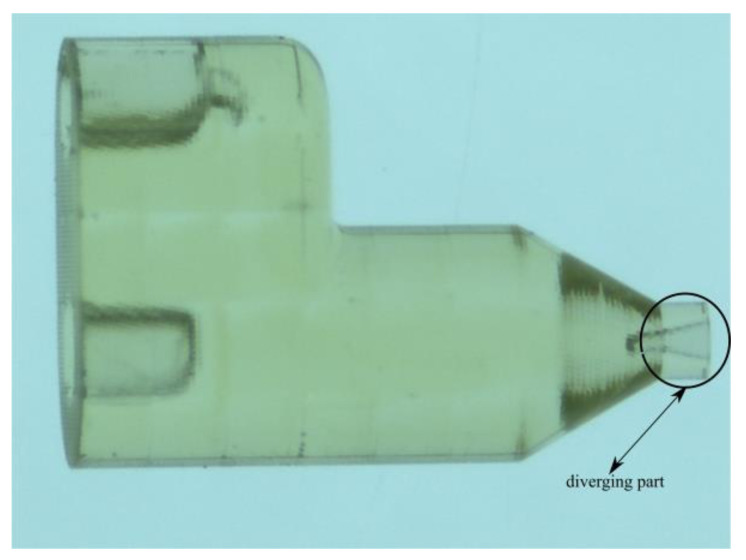
A 3D-printed GDVN with a marked extended diverging outlet orifice.

**Figure 13:**
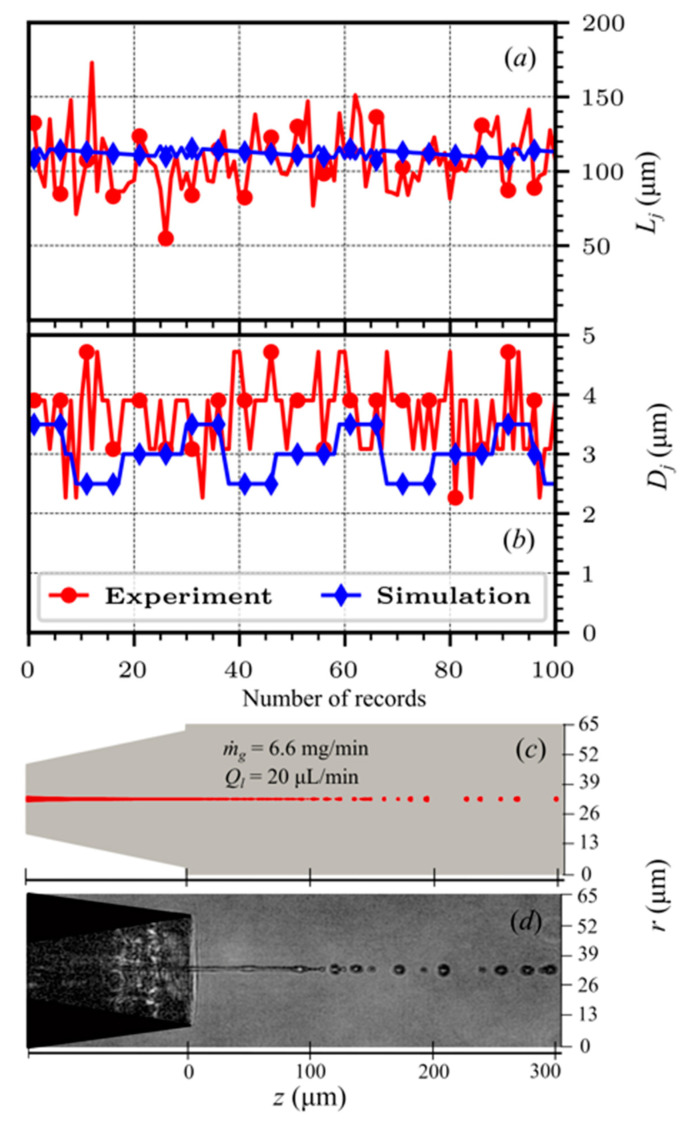
Comparison between the experimentally obtained and the simulated jet shape. The jet (**a**) length and (**b**) diameter over 100 solution records and snapshots from (**c**) numerical simulation and (**d**) experiment. The experimental data were collected every ~2.777 × 10^−5^ s (36,000 frames per second) while in numerical simulation we could track frames every 1.0 × 10^−6^ s. The average values of jet length and diameter from experiments are ~110 µm and ~3.5 µm, whereas from numerical simulations they are ~108 µm and ~3 µm, respectively.

Finally, a converging–diverging nozzle with a diverging length of 130 µm and an angle of 12.5° was prepared using a 3D printer as shown in Figure 12 and was used to focus the liquid jet. The geometric parameters of the printed nozzle matched the diverging angle of the geometric variant 6 in Table 3b, while the length of the diverging (130 µm) part was smaller than the 200 µm used in Table 3b. The nozzle outlet diameter was 118 µm with a throat diameter of 30 µm, the diameter of the liquid feeding capillary was 50 µm, the distance between the liquid outlet and the inner edge of the gas outlet (*H*) was 60 µm and the inner and outer diameters of the gas inlet were 70 and 175 µm, respectively. The jet length and diameter were recovered from the experiment, as explained in [10], and are compared with the results of the present numerical model in Figure 13. The numerical results match well the experimentally measured values of the jet length and diameters produced by the converging–diverging nozzle. This, in addition to the previously reported studies [19,25], provides an additional predictive capability for the converging–diverging nozzles of the numerical model.

The spatial discretization utilized in the numerical model with a cell size of 0.5 µm ensures the jet shape results are within ±0.5 µm. Additionally, the experimental setup utilized also provides the jet shape accuracy within ±0.5 µm [10]. This explains the expected difference in the experimental and the numerical results for the jet diameter in Figure 13.

## 4. Conclusions

This paper provides new insight into the influence of the nozzle outlet orifice designs on the jet length, diameter, and velocity of the GDVN. The study is based on a transient, two-phase, compressible numerical model, solved by the finite volume method and volume of fluid approach. The model was validated in several previous studies. This paper gives an additional validation for one of the new nozzle outlet orifice designs. The nozzle outlet orifice designs were varied in such a way that a converging reference nozzle design was extended by adding (a) constant diameter channels of varying lengths and (b) diverging channels of varying angles at a constant length. The nominal converging nozzle design produced a ~400 µm long, ~6.5 µm thick jet, with a velocity of ~63 m/s.

The extension of the nozzle outlet orifice with constant diameter channels (of length from 0 µm to 250 µm) provides shorter, thinner, and faster jets. The longest channel attached at the outlet orifice provides ~200 µm long, ~5 µm thick jet, with a velocity of ~68 m/s. This jet is ~50% shorter, ~23% thinner, and ~8% faster compared with the nominal converging nozzle design. The converging–diverging arrangement, on the other hand, provides a considerable gain in jet length and jet velocity. According to our study, the nozzle with a diverging angle of 15^o^ and *L_d_* = 200 µm is the most efficient in providing a ~650 µm long and ~4 µm thick jet with a velocity of ~85 m/s. This is ~65% longer, ~35% thinner, and ~35% faster compared with the nominal converging nozzle design. The analysis of the temperature distribution shows no significant change in temperature (~2 K) in the liquid phase near the symmetry axis.

In summary, our simulations predict that a constant diameter extended nozzle outlet orifice design should produce thinner jets. However, to also obtain longer and faster jets one has to use a converging–diverging design with a diverging angle of 15^o^. We demonstrated the usefulness of numerical simulations for testing and optimizing novel nozzle designs and confirmed their validity by comparing them with experimental data for one particular nozzle design.

We demonstrated that changing the nozzle orifice design offers new ways to shape the jet length, diameter, and its velocity. However, the optimum design for a specific application requires a multi-objective design optimization technique that searches for a suitable Pareto front between different design expectations. The design of numerical experiments and the construction of related 3D response surfaces would provide an appropriate starting point for optimization. There are many techniques that could be used to achieve this goal, where combined experimental and model information can be taken into account, such as gray-box modeling [47], evolutionary algorithms [48], or some of the other techniques discussed in a recent review article [49]. 

## Figures and Tables

**Table 1:** An overview of boundary conditions at five patches.

**Patch**	Velocity	Pressure	Temperature	Volume Fraction
Inlet liquid	$Q_{l}{=Q}_{o}$	$\frac{\partial p}{\partial n}=0$	T=293 K	α=1
Inlet gas	${\dot{m}}_{g}={\dot{m}}_{o}$	$\frac{\partial p}{\partial n}=0$	T=293 K	α=0
Walls	no-slip	$\frac{\partial p}{\partial n}=0$	$\frac{\partial T}{\partial n}=0$	$\frac{\partial \alpha }{\partial n}=0$
Outlet	$\frac{\partial v}{\partial n}=0$	p=150 Pa	$\frac{\partial T}{\partial n}=0$	$\frac{\partial \alpha }{\partial n}=0$
Front	wedge *			
Back	wedge *			

* A special boundary condition to enforce the cyclic condition between the two patches.

**Table 2:** Material properties of water and helium at 293 K and 1 bar.

Fluid	ρ (Kgm^−3^)	μ (Pa s)	σ (N/m)	k (W/mK)	$c_{p}$ (kJ/kgK)	$c_{v}$ (kJ/kgK)
H_2_O	1000	1.0 × 10^−3^	0.072	0.58	4.18	4.16
He	0.164	1.9 × 10^−5^	-	0.142	5.19	3.12

**Table 3:** The variants of the nozzle orifice geometry.

(a)			(b)		
*Nozzle*	*L_d_* (µm)	*θ_d_* (°)	*Nozzle*	*L_d_* (µm)	*θ_d_* (°)
1	0	0	6	200	12.500
2	100		7		13.750
3	150		8		14.375
4	200		9		15.000
5	250		10		15.625
			11		16.250
			12		17.500

## Data Availability

The data presented in this study are available on request from the corresponding author.
